# Multi-Case Study on Environmental and Economic Benefits through Co-Burning Refuse-Derived Fuels and Sewage Sludge in Cement Industry

**DOI:** 10.3390/ma15124176

**Published:** 2022-06-13

**Authors:** Karolina Wojtacha-Rychter, Adam Smoliński

**Affiliations:** 1 Central Mining Institute, Department of Mining Aerology, Pl. Gwarków 1, 40-166 Katowice, Poland; kwojtacha@gig.eu; 2 Central Mining Institute, Pl. Gwarków 1, 40-166 Katowice, Poland

**Keywords:** clinker, CO_2_ emissions, RDF, waste, circular economy

## Abstract

The use of waste as an energy source in cement clinker production is a promising way to transition toward a circular economy and limit carbon dioxide (CO_2_) in the atmosphere. The cement industry is responsible for around 5% of global CO_2_ emissions. In this paper, the analysis of environmental and economic profits associated with the substitution of coal by two refuse-derived fuels (RDF) and sewage sludge (SS) in a cement kiln was presented. Differences in the fuel-related CO_2_ emissions were calculated for two-, three-, and four-component fuel blends based on the fuel consumption data, heating values, and the correspondent emission factors. The biogenic fraction content of 19% and 43% were measured in RDFs. The material balance of fuels with the assumed technological parameters of the cement clinker production installation (capacity of 6000 Mg per day and unit heat of 3.6 GJ) shows that the RDF heat substitution at the level of 90% allows for a saving of approximately 28.6 Mg per hour of coal, and to manage even approx. 40 Mg per hour of RDF. The increase in the share of SS in the total heat consumption to 6% contributed to reducing the actual emissions by 17 kg of CO_2_ per 1 Mg of clinker. Multilateral benefits due to the use of RDF in the cement plant were evident.

## 1. Introduction

The cement industry is a model example of the transformation toward a circular economy [1]. A circular economy is a development strategy that means a shift from a linear model, based on the production–consumption–disposal model, to a loop model in which waste becomes a valuable resource. In this solution, the circular economy, thanks to appropriate supply chain management, enables economic growth while optimizing the consumption of natural resources.

The assumptions of the idea of sustainable development and the efficient use of resources are implemented in many cement plants in Poland and around the world through the partial substitution of clinker in cement with raw materials of anthropogenic origin (such as ground blast furnace slag from iron ore production [2,3], fly ash from coal power plants [4], or gypsum waste [5]), and the use of alternative energy sources (i.e., fuel from waste instead of high-emission fossil fuels) [6,7,8,9]. Waste management is one of the most significant challenges of the modern world due to the amount of waste generated and its complex and unstable composition [10,11]. The analysis of data for the period of 1995–2019 [12], concerning changes in the amount of municipal waste generated in the European Union countries and its management, clearly shows an increase in the amount of waste per capita in most countries. In some of them, the increase is up to around 50% (Denmark, Malta). In turn, in France, Belgium, the Netherlands, Germany, Sweden, Great Britain, and Italy, in recent years, stabilization in the amount of generated waste has been observed. Although more waste is generated in the European Union, the total amount of municipal waste landfilled has decreased, while the amount of waste recycled and incinerated has increased significantly. Since 1995, the amount of waste incinerated in the European Union has increased by 30 million Mg or 50% from 30 million Mg (70 kg per capita) in 1995 to 60 million Mg (134 kg per capita) in 2019. In the period of 2010–2019, the amount of waste incinerated increased on average by as much as 5–7% per year.

The decisive factor for the possibility of the thermal processing of waste in cement kilns is its calorific value, determined by the composition of the waste (the share of low and high calorific flammable components), humidity, and the content of non-flammable substances [13,14]. Rezaei et al. [15] revealed that plastic, paper, wood, and organics constitute the major fractions in typical waste fuels used in the cement plant. In the work by [16], the characteristics of these selected fractions found in municipal waste showed that the heating value of plastic at 45 MJ/kg may be three times the heating value of paper and wood at 13 and 16 MJ/kg, respectively. The organic fraction had the lowest heating value at around 7–9 MJ/kg and the highest moisture content at 45–60%. Paper, plastic, and wood had a similar ash content at around 15–16%. The ash content ranged from 3% (wood) to 13% (paper). In addition, the high-temperature (reaching approximately 2000 °C) cement clinker production process and the long stay of exhaust gases at a temperature above 1100 °C (more than 2 s) means that the co-combustion of fuels from waste in the clinker kiln meets the requirements for the thermal waste conversion process and the method of waste management resulting from this process [17]. Moreover, calcium oxide present in the raw material feed neutralizes and captures acid gases such as HCl, HF, and SO_2_ formed during combustion [18]. Ash, a solid residue after combustion, is completely absorbed in the clinker structure, constituting approximately 3.5–4% of its mass [19,20]. Thus, this process allows for both the simultaneous recovery of thermal energy from the organic part of the alternative fuel and material recycling from the mineral part as a valuable component of the raw material set [21]. In cement plants, the alternative fuel is fed through the main burner of the cement kiln, the so-called hot end of the kiln, where the fuel material decomposes in the sintering zone at temperatures of up to 2000 °C [22]. High-calorific waste is mainly fed to the burner, which ensures maintaining a high temperature in the sintering zone and a temperature difference between the combusted material and the flue gases at a level of over 100 °C. Another place where fuels are supplied is the pre-calciner, the so-called secondary burner [23], which is an additional combustion chamber located in the cold part of the rotary kiln, behind the rotary kiln drum and before the cyclone exchanger. Its task is to preheat and calcine the raw material entering the rotary kiln in exchangers [24]. The calciner burns fuels of lower calorific value at a temperature of approximately 1000–1100 °C.

According to the specifications listed in the EN 15375:2011 standard [25], solid fuel, produced from non-hazardous waste and processed through energy recovery in an appropriate installation, is defined as solid recovered fuel (SRF). It is a fuel with set properties that meets the classification and specification requirements of the EN 15359 standard [26]. In turn, in the reference document on the use of the best available techniques in large combustion plants (BREF LCP–Best Available Techniques Reference for Large Combustion Plants), fuels with a sufficiently high calorific value to be used for the combustion or co-combustion with conventional fuels are referred to as secondary fuels [27]. In Polish law [28], the fuel produced from waste is an alternative fuel, which mainly includes refuse derived fuel (RDF) and stabilized sewage sludge, meat and bone meal, waste from the rubber industry, and used tires. The share of RDFs in the alternative fuel consumption structure in Poland is the largest and currently accounts for over 87%. RDFs are a properly sorted and processed combustible, high-calorific fraction of municipal and industrial waste that are recovered from non-hazardous waste, mainly plastics, foil, textiles, paper, and wood.

The cement industry is now facing the pivotal and complex challenges of achieving the goals resulting from the EU Green Deal [29,30] and the requirements set by the Cembureau, the European Cement Producers Association (i.e., achieving carbon neutrality by 2050) [1]. The Cembureau carbon neutrality roadmap demonstrates that reaching net zero emissions along the cement and concrete value chain may be achievable via different pathways. One of the areas of action as part of reducing emissions in the concrete chain is the use of supplementary cementitious materials as a partial substitute of ordinary Portland cement in concrete [31,32]. In turn, one of the options designated by the Cembureau as part of reducing the emissions in the cement chain, is an increase in the waste utilization rate in the energy balance of clinker burning to 60% by 2030 and to 90% by 2050. Until the 1980s, the primary energy carrier in cement plants was coal, which made cement production one of the primary sources of anthropogenic CO_2_ emissions [33]. Currently, this industry is responsible for approximately 5–9% of global CO_2_ emissions [34]. From 2005 to 2019, the average share of heat from alternative fuels in Europe increased from 16% to 50%, and in the cement sector in Poland from 14% to 70% [35]. Several cement plants in Europe and Poland have already achieved 90% of heat substitution from alternative fuels, thanks to the appropriate regulatory environment, social acceptance, and investment support. Examples are the cement plants in Chełm (Poland), operated by Cemex [36]; in Allmendingen (Germany), operated by Schwenk Cement [37]; and in Retznea (Austria), operated by LafargeHolcim [38]. In Poland, in 2021, the average share of heat from alternative fuels in all cement plants was 88% [35,36].

The strategy of increasing the share of heat from alternative fuels in the total heat consumption for cement clinker production will contribute to reducing the impact of cement plants on the environment and local communities by saving primary energy carriers, relieving local landfills and reducing the amount of landfilled waste, or reducing the CO_2_ emissions. Additionally, the substitution of coal with RDFs favors the development of economic activities related to collecting waste and processing it into alternative fuels. The decrease in emissivity results from the fact that alternative fuels have a lower carbon content than conventional fuels. Additionally, they contain a biogenic fraction for which a zero-emission factor is assumed. In Poland, alternative fuels may even contain over 30% of biomass. Den Boer and Jędrczak [39] performed an analysis of the waste input from a mechanical-biological treatment plant in Poland, and detected that the share of biodegradable fractions changed (from 39 to 95%) depending on the size fraction of the studied materials. Nowak et al. [40] found that the concentration of the biodegradable fraction in waste samples classified with 12–19 varied from 25 to 45%. The lowest values were obtained for waste materials with the ash amount of 70% *w*/*w*. The elemental analysis of the RDF samples from several municipal solid waste management plants [41] and a cement plant in Poland [42] showed that the carbon content ranged from 30 to 60% for waste fuels. In comparison, the carbon content in fossil fuels increased to over 90% (for anthracite [43]). Bielowicz et al. [44,45] presented the chemical analysis of the share of elements in 28 coal samples including low-rank and bituminous coal samples derived from the Coal Basin in Poland and showed that the carbon content ranged from 63.40% (lignite) to 89.73% (coking coal).

The assessment of the environmental and economic advantages in terms of the use of RDF and sewage sludge in cement plants was the objective of this paper. The benefits of a cement plant working on coal and partly replacing fossil fuels with an alternative fuel have been presented in many works [43,46,47]. The authors of [43] provided a benefits-based case study where the substitution of 10–90% coal with the alternative fuel was assumed. Kara [47] examined the CO_2_ emissions and petroleum coke savings, when only 10–90% RDF was used as a supplementary fuel. Żygadło and Purgał [46] analyzed the environmental and economic advantages associated with the use of RDF in cement kilns, when the coal was replaced by only 30% of the alternative PAS-r type fuel. In the above-mentioned works, the CO_2_ emissions from fuel combustion were presented mainly for two-component fuel blends (RDF-coal), and the calculation was based on the default emission factors. The literature review revealed that the calculations of the CO_2_ emissions for multi-component fuel blends have not been published thus far. This paper was focused on addressing this knowledge gap. A case study was performed through the analysis of the benefits of two-, and also for three- and four-component fuel blends. Moreover, the calculation of CO_2_ emissions is based on the fuel analysis method, which involves determining the carbon content and calorific value of the fuel combusted. This method is more accurate and preferred for CO_2_ calculations over methods that rely on default emission factors. In the analysis, a variable share of heat flux from two refuse-derived fuels and sewage sludge was included. The amount of fuel being combusted was calculated based on the actual industrial values given by the Polish Cement Association, which collects data from all cement plants in Poland. It was assumed that RDFs of different contents of biogenic fraction were fed into the main burner and in the calciner, in this way, an additional value of this work is introduced. For the assumed technological parameters of the clinker burning installation, the optimal composition of the fuel blend was determined, which will allow for achieving the highest reduction in CO_2_ emissions. In addition, a cost analysis for each fuel configuration proposed was performed to estimate the savings from coal reduction in the fuel blend. The growing interest in the substitution of fossil fuel by waste fuels means that examining the prospects of CO_2_ in the greenhouse gas emissions and energy demand for different fuel configurations may be a valuable source of information for cement producers and a decision support in the CO_2_ emissions reduction policy.

## 2. Materials and Methods

### 2.1. Procedure

There are two main methods to estimate CO_2_ emissions from stationary combustion sources: (1) direct measurement of CO_2_ emissions through the use of a continuous emissions monitoring system and (2) the analysis of fuel input [48]. A case study was performed by the fuel analysis method, which involves determining emissions based on the data on the amount and characteristics of the fuel being consumed [49,50]. This approach is the most widely recommended for CO_2_ calculations because emissions are directly related to the fuel’s carbon content.

In the first step, the heat demand to produce the total mass of clinker was determined. According to the specific heat consumption and cement clinker production data published by the Polish Cement Association in 2021 [36], the total heat demand for the production of the clinker was calculated. Next, basing on the heat demand, the quantitative demand for each fuel type separately was estimated. In this paper, a dry method rotary kiln coupled with a calciner of a production capacity of 6000 Mg of clinker per day and the average specific heat consumption for burning 1 Mg of clinker of 3.6 GJ was surveyed.

Based on the characteristics of the fuel being consumed, the CO_2_ emission factor was determined according to Equation (1):

EF = (44/12∙CC)/Q,(1)

where EF is the carbon emission factor, Mg CO_2_ per terajoule (tCO_2_/TJ); 44/12 is a molar mass ratio of CO_2_ to C; CC is the carbon content, %; Q is the calorific value related to the unit mass, terajoule per Mg.

Next, the fuel-related CO_2_ emissions were calculated according to Equation (2):

E_CO_2__ = M∙EF, (2)

where E_CO_2__ denotes the total CO_2_ emissions from the fuel combustion process, Mg CO_2_, and M is the fuel combusted mass converted into the energy content, TJ per hour. Calculations regarding the fuel-related emissions and savings were carried out in Excel software.

The accuracy of calculating the fuel-related CO_2_ emissions is partially determined by the uncertainty of activity data (i.e., technical errors of fuel weighing) and the uncertainty of the emission factor from the error in determining the calorific value and carbon content of the fuel.

**Figure 1:**
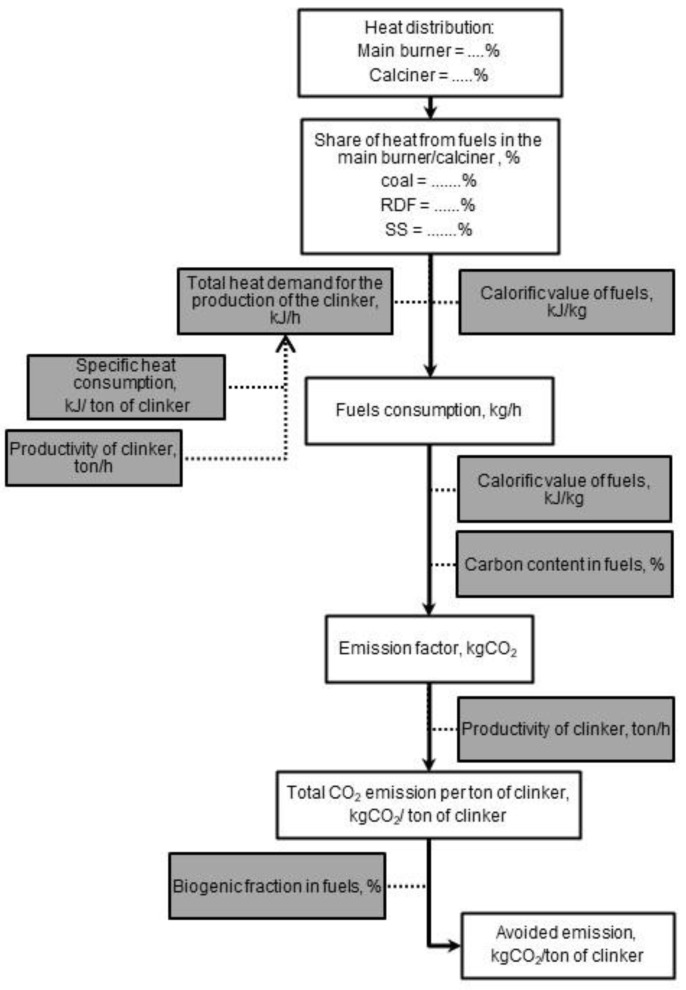
The algorithm to evaluate the CO_2_ emissions from fuel combustion in the cement plant.

The detailed steps of the CO_2_ emissions calculation are illustrated in Figure 1.

In the next stage, the economic benefits of replacing coal by alternative fuels were calculated. The mass saving of the coal and thermal deficit that must be supplemented by the RDF fuel combustion heat was estimated.

### 2.2. Materials

**Figure 2:**
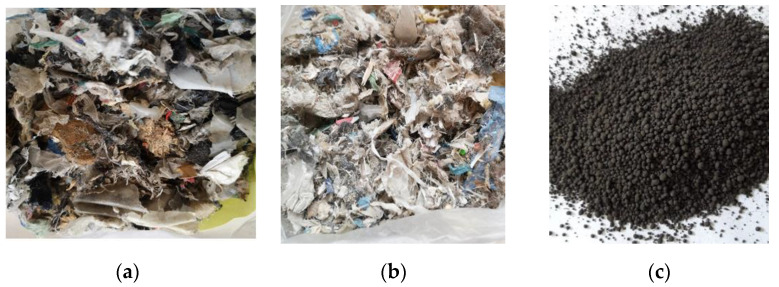
The tested waste sample: (**a**) refuse-derived fuels–RDF 1, (**b**) refuse-derived fuels–RDF 2, and (**c**) sewage sludge–SS.

A multi-case study was performed by the analysis of the effects obtained by the substitution of coal with two refuse-derived fuels (RDF 1 and RDF 2) and sewage sludge (SS) (see Figure 2). To calculate the direct fuel emission, the physical and chemical characteristics of all of the fuels were performed in the accredited Laboratory of Solid Fuels Quality Assessment of the Central Mining Institute. All samples tested were provided by the cement plant (Poland). The RDFs were homogeneous blends of non-hazardous solid waste, which in the Regulation of the Minister of Climate of 2 January 2020 on the catalogue of waste are classified under the code 191210 [28]. Two alternative RDF type fuels are blends made of the over-sieve fraction of waste (80–100 mm), shredded plastic elements, and paper, wood, cardboard, foil, and textiles. In the case of the RDF 1 sample, the fuel was additionally supplemented with rubber chips.

### 2.3. Materials Fuel Blends

Thirty-six fuel blends with different fuel (i.e., coal, RDF 1, RDF 2, and SS) ratios were analyzed to determine the environmental and economic benefits. The percentage of heat from each fuel in the main burner/calciner is presented in Table 1, Table 2 and Table 3. It was assumed that the main burner was fed with a fuel blend consisting of coal and RDF 1, while RDF 2 was only burned in the calciner. The use of low-calorific alternative fuels such as RDF 2 in the main burner was not practiced. The lower heating value of the alternative fuel implied a higher fuel mass flow to maintain the same thermal input, thus increasing the production costs. Moreover, it resulted in a lower gas temperature and a reduction in the energy efficiency of the furnace, resulting in an increase in heat consumption. Sewage sludge is added to the main burner of the cement kiln in the amount of up to 6% of total heat and burned as a fuel. The mass fraction of sludge in the mixtures from 1% to 6% was determined by the equivalent calorific value of the fuel mixtures combusted in the main burner. To obtain the appropriate temperature and shape of the flame in the furnace and to ensure proper heat exchange between the flame and the material on the main burner, the calorific value of the mixture must be more than 22 MJ/kg. Based on the physical-chemical parameters of SS, RDF 1, and coal in Table 4, the equivalent calorific value of the fuel mixtures combusted in the main burner varied from 23 to 28 MJ/kg.

## 3. Results and Discussion

### 3.1. Proximate and Ultimate Analysis

The quality parameters of all of the analyzed fuels are summarized in Table 4. A significant difference between the tested fuels was observed. From the waste results, the highest calorific value was recorded for RDF 1, which was found to have a value of 4.71 MJ/kg lower than that recorded for coal. The calorific value difference between RDF 1 and RDF 2 did not exceed 10 MJ/kg, while between RDF 2 and SS, it was only about 1 MJ/kg. The carbon content in the analyzed fuel materials was at a various level. The highest content of this element was characteristic for the coal, while the lowest for the biomass sample, and the difference between them was 51%. The biogenic fraction in the RDF1 and RDF 2 samples determined by the selective dissolution method was 19.62% and 43.02%, respectively.

### 3.2. CO_2_ Emissions Balance

The emissions balance for each analyzed blend included determining the actual CO_2_ emissions as the sum of unit emissions from the combustion of coal, RDF and sewage sludge, and reduced the CO_2_ emissions. Reduced emissions are the amount of emissions, the balance of which considers the net zero-emission of CO_2_ from the combustion of the biogenic fraction contained in RDF 1, RDF 2, and sewage sludge. The combustion of biomass is considered to be CO_2_ neutral because the emitted amount of the gas is fully offset by the absorption through plant photosynthesis [50]. The results of the fuel emissions for two- component blends in which coal is partially substituted by RDF 1 or sewage sludge heated up in the burner are summarized in Table 5 and Table 6.

As can be seen in Table 5, an increase in the share of heat from RDF 1 by each 10% resulted in a reduction in the actual CO_2_ emissions by about 1.7%. Thus, with the 60% substitution of heat with RDF 1, the actual CO_2_ emissions were lower by about 10% compared to the emissions from fossil fuel combustion alone. Considering the 20% share of biogenic fraction (Table 4) in RDF 1, it resulted in a reduction in the actual CO_2_ emissions by an additional 2–3%. In the final balance, the highest level of CO_2_ emissions reduction was 29%, obtained with the co-combustion of coal with a 90% share of RDF 1 (no. 2a/6) in the total heat.

Based on the analysis of the results presented in Table 6, it was found that the heat substitution from the sewage sludge caused only a slight decrease in CO_2_ emissions, despite its zero-emission factor and low carbon content of 27.78% (Table 4). The highest level of CO_2_ emissions reduction was achieved with the share of heat from sewage sludge at the level of 6% (No. 2b/6). The calculated value of the reduced fuel emissions was only 6% lower than the CO_2_ emissions from coal combustion alone. The low level of CO_2_ emissions reduction for the fuel structure presented in Table 2 resulted from the small share of heat from sewage sludge combustion (1–6%) in the total heat. It is the most unfavorable ecological and economical variant of fuel co-combustion in the clinker burning system among all of the analyzed configurations of fuels. Achieving the level of CO_2_ reduction as with the RDF 1 substitution of coal in the amount of 40–90% (Table 5) would require the share of sewage sludge in the heat balance to be at the level of 13–30%. However, these amounts are very unfavorable for the clinker burning process due to the very low calorific value of sewage sludge, high volatile matter content, and the chemical composition of ash from fuel combustion (e.g., phosphates reduce the raw meal conversion to clinker). Staněka and Sulovskýb [51] studied the effect of phosphorous pentoxide on the phase composition of Portland clinker and found that at 0.7% of phosphorous pentoxide in the clinker, the alite content decreased and belite content declined, while at a phosphorous pentoxide content of 4.5%, the alite formation was totally blocked and the resulting clinker contained free lime. The results of the CO_2_ emissions balance for three-components are summarized in Table 7 and Table 8.

In three-component systems, a greater decline in CO_2_ emissions was obtained by reducing the share of RDF 1 to 50–30% (Nos 3a/4–3a/6) and feeding 40–60% of RDF 2 (Nos. 3a/4–3a/6) than by substituting RDF 1 with 1–6% of sewage sludge in the total heat (Nos. 3b/1–3b/6). From the results in Table 7, when the share of heat from RDF 2 heated up in the calciner increased by 10%, at the expense of the RDF 1 share, the total reduced CO_2_ emissions dropped by 2%. Meanwhile, it was noted that an increase in the share of sewage sludge in the fuel blend by each 1% resulted in reducing the total CO_2_ emissions by less than 1%. When co-combusting coal with RDF 1 and RDF 2 in the amount of 60% and 30% of the total heat (No. 3a/3), respectively, a comparable value of the total reduced CO_2_ emissions was obtained as in the case of 85% of RDF 1 and 5% of sewage sludge in the total heat (No. 3b/5).

An increased share of RDF 2 in the heat structure contributed to the increase in the actual emissions (Table 7), which did not consider the share of the biogenic fraction in the fuel. With a 60% share of heat from RDF 2 (No. 3a/6) heated up in the calciner, the value of actual emissions was 347.13 kg CO_2_ per 1 Mg of clinker, and with a 10% share of heat (No. 3a/1), this value was 320.41 kg CO_2_ per 1 Mg of clinker. Taking into account the 43% share of the biogenic fraction (Table 4) in the RDFs, the total reduced emission value at the level of 235.33 kg CO_2_ per 1 Mg of clinker was obtained. The greater the share of the biogenic fraction in the fuel; the greater reduction in CO_2_ emissions in the total balance. Table 9 and Table 10 present the results of the CO_2_ emissions balance calculated with the assumption of 90% heat substitution from both the RDF 1 and sewage sludge heated up in the burner and from RDF 2 heated up in the calciner.

The calculations made for the four-component blends (Table 9 and Table 10) showed that the values of the reduced CO_2_ emissions were at a comparable level. It showed that the substitution of RDF 1 with the sludge in the main burner had the same CO_2_ reduction effect as the substitution of RDF 2 with the sludge in the calciner. The substitution of both RDF 1 and RDF 2 with sewage sludge by each 1% lowered the reduced emissions by only 2 kg of CO_2_ per 1 Mg of clinker.

**Figure 3:**
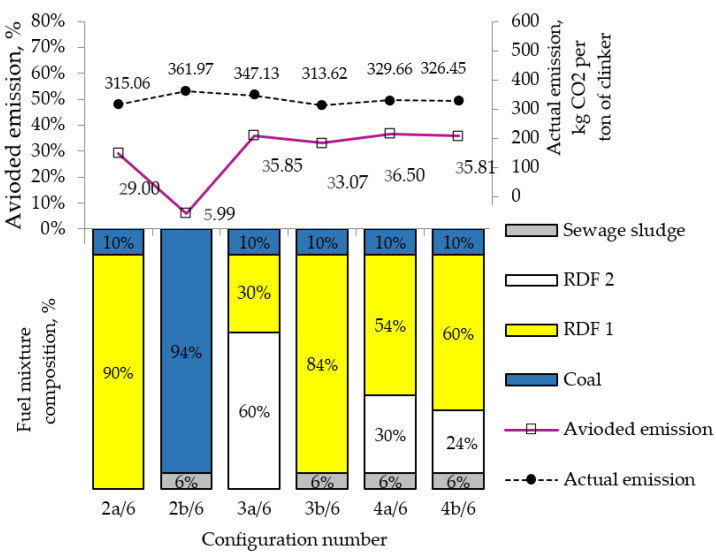
The comparison of the level of CO_2_ reduction for the different co-combustion configurations of coal with RDFs or sewage sludge (SS).

Figure 3 summarizes the highest levels of avoided CO_2_ emissions achieved in each fuel configuration in two-, three-, and four-component blends, the actual amount of CO_2_ emissions per 1 Mg of clinker, and the share of heat from individual fuels. The level of emissions reduction (%) as a result of the partial replacement of coal with fuels containing the biogenic fraction was calculated concerning the emissions level of 366.86 kg of CO_2_ per 1 Mg of clinker (i.e., with 100% coal combustion with the quality parameters given in Table 5).

The highest level of avoided CO_2_ emissions (36.50%) was obtained for the fuel configuration (No. 4a/6) (i.e., for coal substitution with RDFs and sewage sludge) at a total of 90% including a 30% share of heat from RDF 2 and 6% share of heat from sewage sludge (see Figure 3). It was found that the level of avoided CO_2_ emissions for configuration No. 3a/6 (i.e., 30% and 60% share of heat from RDF 1 and RDF 2, respectively) is comparable to the level of CO_2_ reduction achieved for configuration No. 4b/6 (i.e., 60%, 24%, and 6% in the share of heat from RDF 1, RDF 2, and sewage sludge, respectively). The increase in the share of heat from RDF 1 heated up in the burner to over 80% (No. 2a/6 and No. 3b/6) resulted in the actual CO_2_ emissions reduction by 14–15% in comparison with the emissions from the combustion of coal alone in the clinker burning system (see Figure 3). With the 50–60% and about 30% substitution of coal with RDF 1 and RDF 2 fuels (Nos. 4a/6 and 4b/6), respectively, the actual CO_2_ emissions were lower only by approximately 10–11%. For comparison, the actual emission level for fuel variant No. 3a/6 was reduced by only 5%, reaching the value of 347.13 kg CO_2_ per 1 Mg of clinker. The performed calculation showed that the CO_2_ emissions from fossil fuels in a cement plant may decrease by more than 35% with an appropriately selected heat structure from individual fuels.

### 3.3. Material and Economic Balance

**Figure 4:**
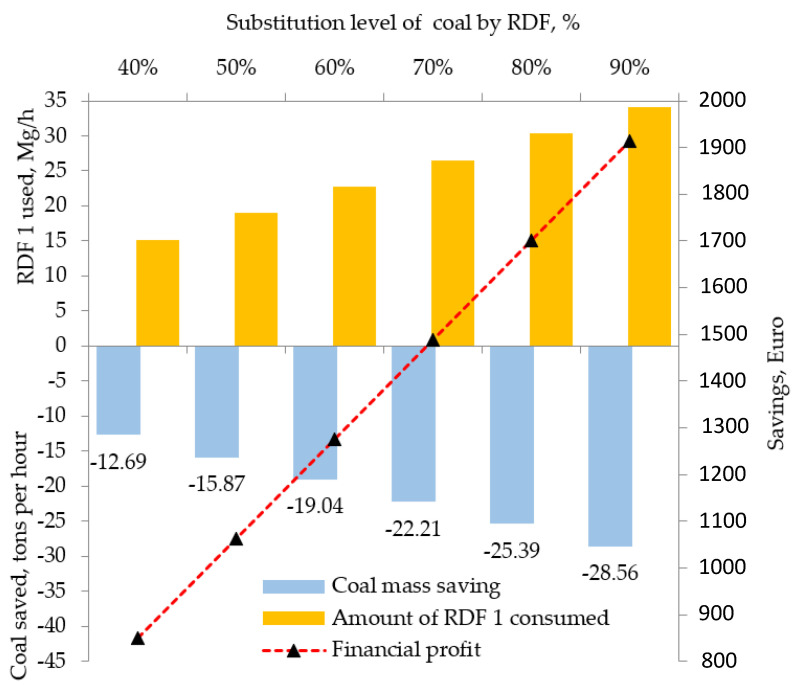
The coal and RDF 1 consumption balance for two- component blends (Table 1).

For the fuel configuration No. 2a/6 in Table 1, in which the primary energy carrier is partially substituted with RDF 1, 40% to 90% of the total heat consumption, the financial profit from conventional fuel savings, and the hourly demand for RDF 1 to cover the thermal deficit fully were calculated. The calculation results are shown in Figure 4.

According to the assumed production data, the total heat demand to produce 250 Mg of clinker per hour was 912,500 MJ. The amount of coal of calorific value of 28.76 MJ/kg necessary to obtain such heat was 32 Mg per hour. For comparison, the quantitative demand for RDF 1 to obtain the same amount of heat was 38 Mg per hour, and for RDF 2, it was almost twice as high (i.e., 65 Mg per hour). The thermal deficit caused by a 90% reduction in coal in the total heat was 821,250 MJ and should be supplemented with about 34.2 Mg per hour of RDF 1 with the calorific value of 24.05 MJ/kg (i.e., 5.6 Mg per hour more fuel from waste than coal must be fed into the burner, see Figure 4). The lower the calorific value of RDF, the greater the fuel feed to the clinker burning system, which requires a continuous supply of RDFs or larger storage areas. Each 10% increase in the share of heat from RDF 1 saves 3.2 Mg of coal per hour, which gives a financial profit of EUR 213 (see Figure 4). In the work by [40], the mass saving of the coal with the assumption of 30% RDF combustion was equal to 3 Mg per hour. The savings due to lower coal consumption were calculated by assuming that the purchase cost of fossil fuel substituted with RDF is EUR 67 per 1 Mg [52]. The actual financial profit from the partial substitution of conventional fuel with RDF will be higher because the calculation does not consider the ability of the cement plants to receive waste disposal fees. In Poland, these amounts may vary from about EUR 20 to EUR 40 per 1 Mg of alternative fuel, depending on the quality parameters of the fuel [53,54]. The total economic effect of co-combusting alternative fuels in the clinker burning system consists of the current relationship between the price of coal and the price of alternative fuel on the market and the prices of the CO_2_ emissions allowances. In the analyzed case, each 10% increase in the share of heat from the alternative fuel, RDF 1, means about 11.82 kg of CO_2_ emissions less per 1 Mg of clinker compared to the emissions level during the combustion of 100% coal in the system. In 2021, the prices of the EU’s CO_2_ emissions allowances broke new historical records. In January, according to the European Union’s Emissions Trading System (EU ETS) [55], the prices of the allowances reached a level close to EUR 35, and in mid-March, they exceeded EUR 40. Currently, for the right to emit 1 Mg of this essential greenhouse gas, for the first time, it was necessary to pay a record value of about EUR 52 per 1 Mg. At such a price of CO_2_ emissions allowances, the financial profit obtained due to the 10% substitution of coal with RDF 1 with the share of the CO_2_ emissions from neutral biomass amounted to EUR 615 per 1 Mg of clinker. At a higher share of RDF 1 of 90% in the total heat consumption, the profit from the avoided emissions would amount to EUR 5534 per 1 Mg of clinker. The total financial gain for the six selected configurations (see Figure 3) with the lowest value of reduced CO_2_ emissions is presented in Table 11.

The calculations were made based on the material balance presented in Table 12, which clearly shows that the consumption of individual fuels in the six considered configurations was diversified. The calculation was based on the gate fee for the high-calorific RDF 1 of EUR 33 per Mg, and for the RDF 2 used in the calciner and sewage sludge of EUR 22 per Mg.

The list presented in Table 11 shows that the best financial results were achieved for fuel configuration Nos. 3a/6 and 4a/6. This is due to the fact they both achieved high levels of CO_2_ reduction and the consumption of RDFs was about 40–50 Mg per hour for the fuel variants. The results in Table 11 also indicate that the profit obtained from avoided CO_2_ emissions had the most significant impact on the final financial balance.

## 4. Conclusions

1. The performed calculations showed that the change in the energy carrier in the clinker burning system from fossil fuel (coal) to waste derived fuel containing the biomass fraction is highly profitable for the cement industry. This will contribute to the reduction in fuel-related CO_2_ emissions and the savings of more expensive coal.
2. It was found that the CO_2_ emissions per mass of cement clinker production varied significantly among the compositions of the different fuel mixtures. It was observed that the introduction of low calorific fuel to the calciner increased the reduction in CO_2_ emissions by an additional 3%.
3. The highest CO_2_ emissions reduction (from 366.86 kg of CO_2_ per 1 Mg of clinker to 233–257 kg of CO_2_ per 1 Mg of clinker) and the reduction in the production cost were obtained by working on the four-component mixture by substitution of 90% coal with 54% of RDF 1, 30% RDF 2, and 6% sewage sludge.
4. The increase in the amount of heat from the sewage sludge, which was considered neutral in terms of CO_2_ emissions, to 6%, reduced the CO_2_ emissions. However, in relation to the total emissions balance, their share of 2–17 kg CO_2_ per 1 Mg of clinker was insignificant. Co-combustion of coal with 6% of sewage sludge and RDFs containing the biomass fraction burnt in the calciner and the main burner at the level of 30% and approximately 60% of the total heat, respectively, turned out to be the most beneficial from the point of view of climate protection. A comparable level of reduction was also obtained in the co-combustion of coal with RDFs alone, but with two times the higher share of RDFs containing biomass fractions at the level of 43%.
5. The economic balance showed that increasing the share of energy from waste in the fuel structure in the clinker firing system contributes to the reduction in production costs due to lower CO_2_ emission fees, gate fees for cement plants, and the reduction in the consumption of more expensive conventional fuels.

The environmental benefit of replacing fossil fuels with alternative fuels in the cement industry also means eliminating millions of Mg of waste that cannot be recycled from the environment while preserving the reserves of fossil fuels. The results of the study confirm that the substitution of coal with waste fuel in the cement industry is one of the directions of activities supporting the implementation of the goals of the European Green Deal and the idea of a circular economy. However, this work had some limitations that can be improved upon in the future. This calculation only describes the benefits for cement producers, which was obtained by using the two selected refuse-derived fuels and sewage sludge. As is known, the problem related to the use of alternative fuels derived from waste is its heterogeneity, and thus the variability in the emissions factor. In the future, it is also worth carrying out a comparative study of CO_2_ emissions from the co-firing of gaseous fuel, because a big potential for CO_2_ reduction also lies in replacing coal with natural gas.

## Figures and Tables

**Table 1:** The heat distribution in the two-components blends, (in %).

No.	Coal	RDF 1	SS	RDF 2	
	Main Burner			Calciner	
2a/1	60	40	0	0	It was assumed that the required amount of heat needed to burn the clinker is obtained from the co-combustion of coal with RDF 1 in the main burner. The RDF 1 share varied from 40 to 90%.
2a/2	50	50	0	0	
2a/3	40	60	0	0	
2a/4	30	70	0	0	
2a/5	20	80	0	0	
2a/6	10	90	0	0	
2b/1	99	0	1	0	It was assumed that the required amount of heat needed to burn the clinker is obtained from the co-combustion of coal with SS in the main burner. The SS share varied from 1 to 6%.
2b/2	98	0	2	0	
2b/3	97	0	3	0	
2b/4	96	0	4	0	
2b/5	95	0	5	0	
2b/6	94	0	6	0	

**Table 2:** The heat distribution in the three-component blends, (in %).

No.	Coal	RDF 1	SS	RDF 2	
	Main Burner			Calciner	
3a/1	10	80	0	10	It was assumed a constant share of heat from coal. The remaining 90% of the heat was obtained from combustion of RDF 1 in the main burner and RDF 2 in a calciner. The RDF 1 and RDF 2 shares varied from 30 to 80% and from 10 to 60%, respectively.
3a/2	10	70	0	20	
3a/3	10	60	0	30	
3a/4	10	50	0	40	
3a/5	10	40	0	50	
3a/6	10	30	0	60	
3b/1	10	89	1	0	It was assumed a constant share of heat from coal. The remaining 90% of the heat was obtained from co-combustion of RDF 1 and SS in a main burner. The RDF 1 and SS shares varied from 84 to 89% and from 1 to 6%, respectively.
3b/2	10	88	2	0	
3b/3	10	87	3	0	
3b/4	10	86	4	0	
3b/5	10	85	5	0	
3b/6	10	84	6	0	

**Table 3:** The heat distribution in the four-component blends, (in %).

No.	Coal	RDF 1	SS	RDF 2	
	Main Burner			Calciner	
4a/1	10	59	1	30	It was assumed a constant share of heat from coal and RDF 2. The remaining 60% of the heat was obtained from combustion RDF 1 and SS in the main burner. The RDF 1 and SS share varied from 54 to 59% and from 1 to 6%, respectively.
4a/2	10	58	2	30	
4a/3	10	57	3	30	
4a/4	10	56	4	30	
4a/5	10	55	5	30	
4a/6	10	54	6	30	
4b/1	10	60	1	29	It was assumed a constant share of heat from coal and RDF 1. The remaining 60% of the heat was obtained from combustion RDF 2 and SS in the main burner. The RDF 2 and SS share varied from 24 to 29% and from 1 to 6%, respectively.
4b/2	10	60	2	28	
4b/3	10	60	3	27	
4b/4	10	60	4	26	
4b/5	10	60	5	25	
4b/6	10	60	6	24	

**Table 4:** The physicochemical parameters of the tested fuels.

Parameter	Coal	RDF 1	RDF 2	SS
Proximate analysis (wt.%)				
Moisture	5.64	13.07	7.21	5.63
Ash	8.90	7.84	20.04	34.41
Volatiles	32.61	80.75	64.25	93.15
Elemental analysis (wt.%)				
Carbon	78.88	55.62	38.11	27.78
Hydrogen	4.80	8.42	5.15	4.34
Nitrogen	1.22	0.47	1.40	5.80
Sulfur	0.36	0.13	0.04	1.43
Oxygen	14.74	35.36	55.30	60.65
Heat combustion, MJ/kg	31.26	29.29	15.23	14.19
Calorific Value, MJ/kg	28.76	24.05	14.05	13.02

**Table 5:** The CO_2_ emissions balance of blends of coal with RDF 1 (2a—two components).

No.	Emissions				Actual Fuel Emissions	Total Reduced Fuel Emissions
	Coal	RDF 1	SS	RDF 2		
	kg CO_2_/Mg of Clinker					
2a/1	220.12	123.72	-	-	343.84	319.56
2a/2	183.43	154.65	-	-	338.08	307.74
2a/3	146.74	185.58	-	-	332.32	295.91
2a/4	110.06	216.51	-	-	326.57	284.09
2a/5	73.37	247.44	-	-	320.81	272.27
2a/6	36.69	278.37	-	-	315.06	260.44

**Table 6:** The CO_2_ emissions balance of blends of coal with SS (2b—two components).

No.	Emissions				Actual Fuel Emissions	Total Reduced Fuel Emissions
	Coal	RDF 1	SS	RDF 2		
	kg CO_2_/Mg of Clinker					
2b/1	363.19	-	2.85	-	366.04	363.19
2b/2	359.52	-	5.71	-	365.23	359.52
2b/3	355.86	-	8.56	--	364.42	355.86
2b/4	352.19	-	11.42	-	363.61	352.19
2b/5	348.52	-	14.27	-	362.79	348.52
2b/6	344.85	-	17.12	-	361.97	344.85

**Table 7:** The CO_2_ emissions balance of blends of coal with RDF 1-RDF 2 (3a—three components).

No.	Emissions				Actual Fuel Emissions	Total Reduced Fuel Emissions
	Coal	RDF 1	SS	RDF 2		
	kg CO_2_/Mg of Clinker					
3a/1	36.69	247.44	-	36.28	320.41	256.26
3a/2	36.69	216.51	-	72.55	325.75	252.07
3a/3	36.69	185.58	-	108.83	331.10	247.89
3a/4	36.69	154.65	-	145.10	336.44	243.70
3a/5	36.69	123.72	-	181.38	341.79	239.52
3a/6	36.69	92.79	-	217.65	347.13	235.33

**Table 8:** The CO_2_ emissions balance of blends of coal with RDF 1-SS (3b—three components).

No.	Emissions				Actual Fuel Emissions	Total Reduced Fuel Emissions
	Coal	RDF 1	SS	RDF 2		
	kg CO_2_/Mg of Clinker					
3b/1	36.69	275.28	2.85	-	314.82	257.95
3b/2	36.69	272.19	5.71	-	314.59	255.47
3b/3	36.69	269.09	8.56	-	314.34	252.98
3b/4	36.69	266.00	11.42	-	314.11	250.50
3b/5	36.69	262.91	14.27	-	313.87	248.01
3b/6	36.69	259.81	17.12	-	313.62	245.52

**Table 9:** The CO_2_ emissions balance of blends of coal with RDF 1-RDF 2-SS (4a—four components).

No.	Emissions				Actual Fuel Emissions	Total Reduced Fuel Emissions
	Coal	RDF 1	SS	RDF 2		
	kg CO_2_/Mg of Clinker					
4a/1	36.69	182.49	2.85	108.83	330.86	245.40
4a/2	36.69	179.39	5.71	108.83	330.62	242.91
4a/3	36.69	176.30	8.56	108.83	330.38	240.43
4a/4	36.69	173.21	11.42	108.83	330.15	237.94
4a/5	36.69	170.12	14.27	108.83	329.91	235.46
4a/6	36.69	167.02	17.12	108.83	329.66	232.97

**Table 10:** The CO_2_ emissions balance of blends of coal with RDF 1-RDF 2-SS (4b–four components).

No.	Emissions				Actual Fuel Emissions	Total Reduced Fuel Emissions
	Coal	RDF 1	SS	RDF 2		
	kg CO_2_/Mg of Clinker					
4b/1	36.69	185.58	2.85	105.20	330.32	245.82
4b/2	36.69	185.58	5.71	101.57	329.55	243.75
4b/3	36.69	185.58	8.56	97.94	328.77	241.68
4b/4	36.69	185.58	11.42	94.32	328.01	239.62
4b/5	36.69	185.58	14.27	90.69	327.23	237.55
4b/6	36.69	185.58	17.12	87.06	326.45	235.48

**Table 11:** The economic balance.

No.	Financial Profit from Avoided CO_2_ Emissions, EUR/h	Financial Profit from Saving Coal, EUR/h	Financial Profit from RDF and SS Gate Fees, EUR/h	Total Profit, EUR/h
2a/6	5534	1914	1138	8586
2b/6	1145	128	94	1367
3a/6	6839	1914	1244	9997
3b/6	6309	1914	1156	9379
4a/6	6962	1914	1209	10,085
4b/6	6832	1914	1199	9945

**Table 12:** The fuel consumption balance.

No.	RDF 1, Mg/h	RDF 2, Mg/h	SS, Mg/h	Coal, Mg/h
2a/6	34.14	0	0	28.56
2b/6	0	0	4.21	1.91
3a/6	11.34	38.97	0	28.56
3b/6	31.87	0	4.21	28.56
4a/6	20.49	19.48	4.21	28.56
4b/6	22.77	15.59	4.21	28.56

## Data Availability

Not applicable.
